# Case Report: Diazoxide-induced diabetic ketoacidosis in a patient with insulinoma

**DOI:** 10.3389/fendo.2025.1524288

**Published:** 2025-05-01

**Authors:** Run Ting Chin, Jolene Jiayu Kiew, Khek Yu Ho, Doddabele S. Deepak

**Affiliations:** ^1^ Division of Endocrinology, Department of Medicine, National University Hospital, National University Health Systems, Singapore, Singapore; ^2^ Division of Endocrinology, Department of Medicine, Ng Teng Fong General Hospital, National University Health Systems, Singapore, Singapore; ^3^ Division of Gastroenterology and Hepatology, Department of Medicine, National University Hospital, National University Health Systems, Singapore, Singapore

**Keywords:** insulinoma, diazoxide, diabetic ketoacidosis, endoscopic ultrasound, radiofrequency ablation

## Abstract

Definitive management of insulinoma is challenging as curative surgery is associated with a considerable risk of morbidity and mortality. The successful long-term use of diazoxide for patients diagnosed with insulinoma has been described, but rarely, the use of diazoxide can be complicated by diabetic ketoacidosis (DKA). This case report describes a frail elderly patient with high surgical risk diagnosed with pancreatic head insulinoma whose initial treatment with diazoxide was complicated by a hyperglycemic crisis. Her insulinoma was eventually managed with endoscopic ultrasound-guided-radiofrequency ablation (EUS-RFA) with complete resolution of hypoglycemic episodes. This case highlights the importance of monitoring for side effects of diazoxide, especially in the elderly, and the role of EUS-RFA as an emerging non-surgical treatment modality for insulinoma.

## 1 Introduction

Insulinomas are extremely rare pancreatic endocrine tumors with an annual incidence of four cases per million people in the general population (1). Most insulinomas are sporadic, solitary, and benign, yet they are associated with life-threatening hypoglycemia due to autonomous insulin secretion (2). The diagnosis is often delayed or missed due to the rarity of insulinomas and non-specific symptoms. The presence of a hypoglycemic disorder is established by satisfying Whipple’s triad – documented low blood glucose, in presence of clinical symptoms consistent with hypoglycaemia, and prompt relief of symptoms after plasma glucose is raised. This case illustrates the challenges associated with the diagnosis of insulinoma in an elderly patient with cognitive impairment. More importantly, it also highlights the need for increased vigilance for potential side effects of diazoxide, especially in elderly patients with impaired renal function. There are only a few case reports (3–5) on hyperglycemic crises occurring following diazoxide therapy. These tend to occur in elderly patients in the setting of other contributing factors including dehydration, infection, or new drugs (such as steroids), and even occur after many years of being on diazoxide (6).

In view of the patient’s advanced age and comorbidities precluding major pancreatic surgery, her insulinoma was eventually managed with endoscopic ultrasound-guided-radiofrequency ablation (EUS-RFA) with complete resolution of hypoglycemic episodes. This case adds to the evolving evidence of RFA being a potential safe and feasible therapeutic modality for patients who refuse surgery (7) or selected patients with benign insulinoma who are at high surgical risk.

## 2 Clinical presentation

A 94-year-old woman was brought into the hospital via ambulance following an unwitnessed fall at home. The result of the point-of-care testing (POCT) of glucose performed on-site was 3.3 mmol/L. The patient complained of giddiness and was noted to be slightly sweaty. Due to her background of dementia, a corroborative history from her son was sought. He reported behavior changes over the past 1 month. The patient was noted to be more sleepy and “became very violent in her sleep when she would kick the cabinet, wave her limbs or struggle in her sleep”.

There were no episodes of diaphoresis or impaired consciousness and no recent change in her weight. Her appetite was mood-dependent and variable. She had taken over-the-counter calcium and fish oil supplements for the past 1 year. There were no other new medications, health supplements, or traditional medication used. No one at home used any insulin or oral glucose lowering agents (oGL) at that time.

Her past medical history included hypertension, hyperlipidemia, diagnosed Alzheimer’s dementia since 2017, adjustment disorder with depressed mood, post-menopausal osteoporosis, and chronic kidney disease (CKD). She required assistance in basic activities of daily living, with short-term memory loss for more than 10 years.

On arrival at the hospital, she was alert but not oriented to time, place, or person. Her blood pressure was 160/84mmHg, and her pulse rate was 68 beats per minute. She was afebrile, saturating at 100% on room air with a respiratory rate of 20 breaths per minute. Her weight was 35.1kg, with a body mass index of 19.3kg/m^2^. She had no focal neurological deficits. Cardiovascular, respiratory, and abdominal examinations were unremarkable.

Initial investigations were notable for low venous blood glucose of 2.2 mmol/L and hypokalemia (serum potassium of 3.1mmol/L). She had otherwise normal full blood count and liver function and had stable chronic kidney disease with a calculated creatinine clearance of 28ml/min. Computed tomography (CT) of the brain was unremarkable. She continued to have multiple episodes of hypoglycemia on POCT glucose monitoring over the subsequent 2 days of hospitalization.

With a background of dementia in this elderly patient, eliciting Whipple’s triad was difficult as she did not complain of typical hypoglycemic symptoms. In view of the clinically significant spontaneous hypoglycemia episodes with capillary blood glucose (CBG) as low as 1.6 mmol/L, further evaluations with venous glucose, serum insulin, serum C-peptide, and beta-hydroxybutyrate were conducted when capillary glucose was < 3mmol/L (**Table 1**). Her 8 am cortisol was 309nmol/L, with a peak cortisol of 618nmol/L at 60 minutes after Synacthen administration. Her thyroid function test was normal [fT4 9.7pmol/L (ref 8.0-16.0) and Thyroid-stimulating hormone (TSH) 0.77mIU/L (ref 0.45 – 4.50)].

**Table 1:** Results showing endogenous hyperinsulinemic hypoglycemia.

	1^st^ episode	2^nd^ episode	Unit	Reference interval
Point-of-care testing of glucose	2.0	3.1	mmol/L	3.0 – 6.0
Venous glucose	1.3	2.0	mmol/L	3.0 – 6.0
B-hydroxybutyrate	<0.6	<0.6	mmol/L	<0.6
Insulin	7.9	12.0	mU/L	<3.0
C-peptide	1193	1186	pmol/L	<200

Biochemistry tests during two spontaneous hypoglycemia episodes confirmed endogenous hyperinsulinemic hypoglycemia (low serum glucose levels, high serum insulin, and high serum C-peptide with a low β-hydroxybutyrate) (**Table 1**). A toxicological screen for insulin secretagogue use and insulin autoimmune syndrome returned negative. The differential diagnoses included insulinoma or non-insulinoma pancreatogenous hypoglycemia syndrome (NIPHS). CT of the pancreas revealed a normal appearance and enhancement of the pancreas with no focal lesions or pancreatic duct dilatation.

## 3 Management

The patient was started on regular oral nutritional supplements in view of her mildly malnourished status and poor oral intake. Despite this, she continued to have recurrent hypoglycemia episodes and required two liters per day of IV dextrose 10% to maintain her blood glucose above 4 mmol/L.

Diazoxide was started and titrated to 100mg twice daily, which allowed weaning of the dextrose infusion. Her capillary blood glucose was in the range of 4.1mmol/L to 10.7mmol/L for the subsequent 3 days. She was discharged well on diazoxide 100mg twice daily, with a plan for clinical review in 3 weeks’ time. Her son was educated regarding home glucose monitoring and management of hypoglycemia.

At the clinical review, she was noted to be adherent to diazoxide with no recurrence of hypoglycemia (home glucose monitoring between 9 to 20mmol/L). However, the patient had become more lethargic and confused over the past few weeks, with reduced oral intake. Her capillary blood glucose test conducted in the clinic was elevated at 29.1mmol/L. Subsequent investigations (**Table 2**) revealed that she was in diabetic ketoacidosis (DKA) and a hyperglycemic hyperosmolar state (HHS) precipitated by diazoxide use. She was started on intravenous insulin infusion, and transitioned to subcutaneous insulin 16 hours later. The half-life of diazoxide varies between 24 to 36 hours, which may be further prolonged in the presence of renal impairment as it has renal excretion. Considerations of the prolonged half-life of diazoxide have also contributed to our decision for transition from intravenous insulin to subcutaneous insulin. In view of poor oral intake with borderline low blood glucose (ranging 4 to 6mmol/L), subcutaneous insulin was entirely discontinued the following day.

**Table 2:** Results showing diabetic ketoacidosis and a hyperglycemic hyperosmolar state precipitated by diazoxide use.

Investigation	Result	Units	Reference interval
Sodium	155	mmol/L	135 – 145
Potassium	3.7	mmol/L	3.5 – 5.0
Carbon dioxide	6	mmol/L	22 – 31
Creatinine	140	umol/L	50 – 90
Urea	8.9	mmol/L	2.0 – 6.5
Glucose	34.8	mmol/L	3.0 – 6.0
Anion Gap	34	mmol/L	
estimated glomerular filtration rate (eGFR)	28	ml/min	
B-hydroxybutyrate	5.7	mmol/L	

Thereafter, the hypoglycemic episodes recurred, again necessitating intravenous dextrose infusion. In view of the previous hyperglycemic crisis precipitated by diazoxide, she was started on oral prednisolone, which was eventually increased to 2.5mg twice daily. Despite this, she continued having hypoglycemic episodes with CBG as low as 1.3mmol/L.

### 3.1 Localization of insulinoma

**Figure 1:**
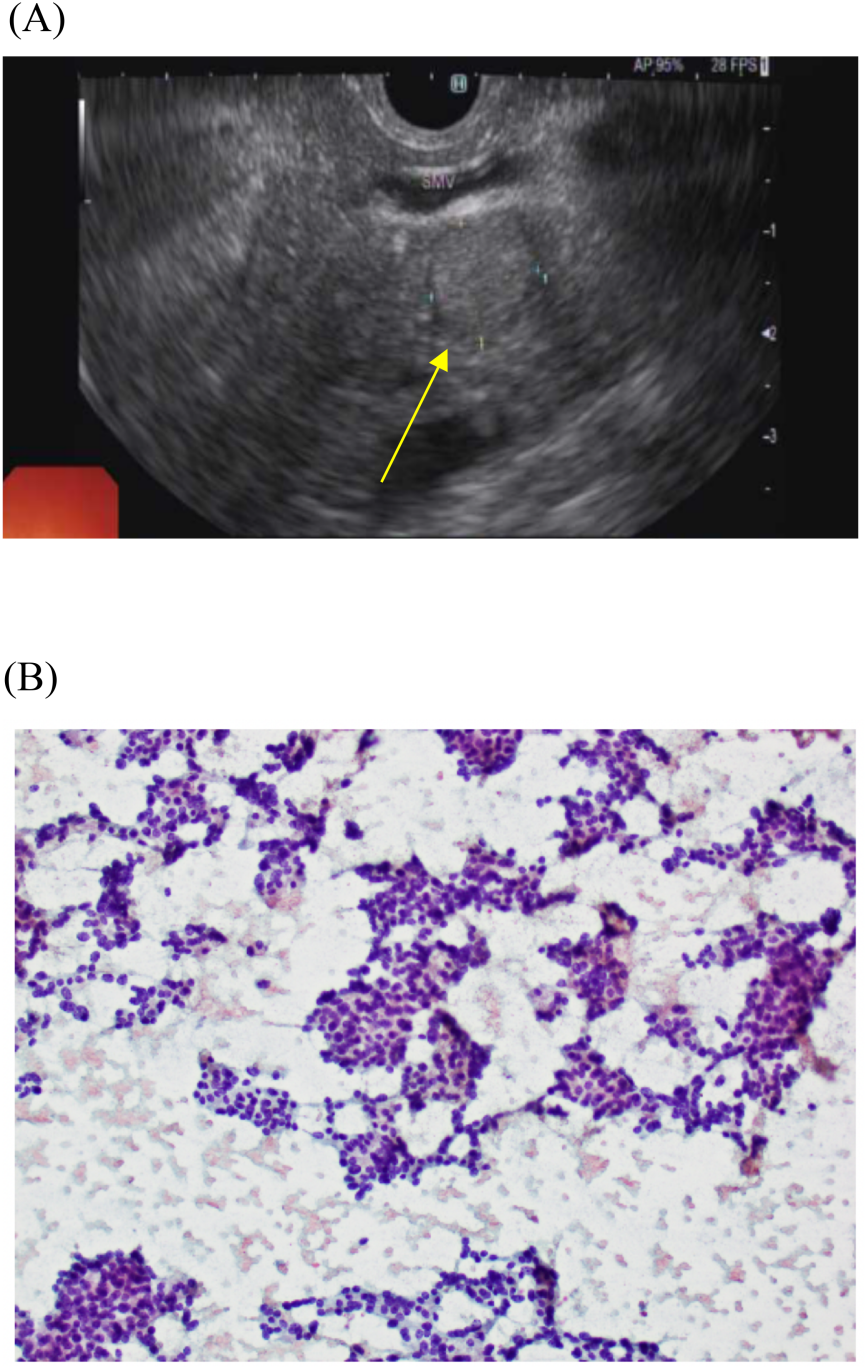
**(A)** Image showing mass in head of pancreas taken during an endoscopic ultrasound for localization of insulinoma. **(B)** Histopathological slide from endoscopic ultrasound-guided fine needle aspiration of pancreatic head insulinoma, showing nests of uniform cells.

The patient underwent an endoscopic ultrasound (EUS), which identified a 1.4 cm × 1.2 cm well-defined hyperechoic rounded mass in the head of pancreas, with an adjacent focus of calcification (**Figure 1**). The rest of the pancreas looked atrophied. Fine needle aspiration (FNA) and biopsy of the mass were performed.

Histopathology of the core biopsy obtained from the head of the pancreatic mass showed features consistent with a neuroendocrine tumor (**Figure 1**), confirming the diagnosis of a pancreatic head insulinoma. Sections showed fragmented core biopsies comprising some nests and small sheets of relatively uniform cells with round to oval nuclei, stippled chromatin, and variable amounts of eosinophilic cytoplasm. Immunohistochemistry revealed that most of the lesional cells showed strong reactivity with synaptophysin and there is no appreciable reactivity with chromogranin.

### 3.2 Definitive management of insulinoma

**Figure 2:**
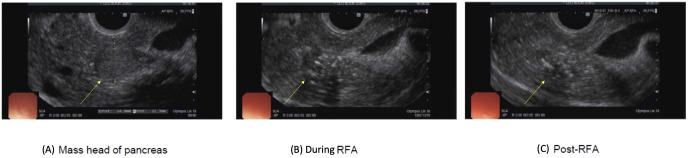
Images taken during endoscopic ultrasound (EUS) and radiofrequency ablation (RFA) showing **(A)** a hypoechoic lesion in the head of the pancreas. EUS image of the lesion **(B)** during the RFA and **(C)** after the RFA. A total of 4 ablations (30W of 10 seconds each) were conducted into the tumor.

After extensive discussion with gastroenterology colleagues and patient’s family, EUS-RFA (**Figure 2**) was deemed to be the most suitable and potentially definitive therapy for this patient – given her high surgical risk as well as major adverse event from use of first line medical therapy with diazoxide. There was a notable improvement in her glycaemic trend within one day of EUS-RFA, allowing for complete discontinuation of IV dextrose infusion and oral prednisolone five days later while maintaining normoglycemia.

In the period leading up to radiofrequency ablation, she was having recurrent hypokalaemia requiring multiple cycles of intravenous potassium replacement. Hypokalemia was likely due to intracellular shifts from hyperinsulinemia and contributed by refeeding. The patient was started on intravenous and oral potassium replacement as required to maintain normokalemia.

**Figure 3:**
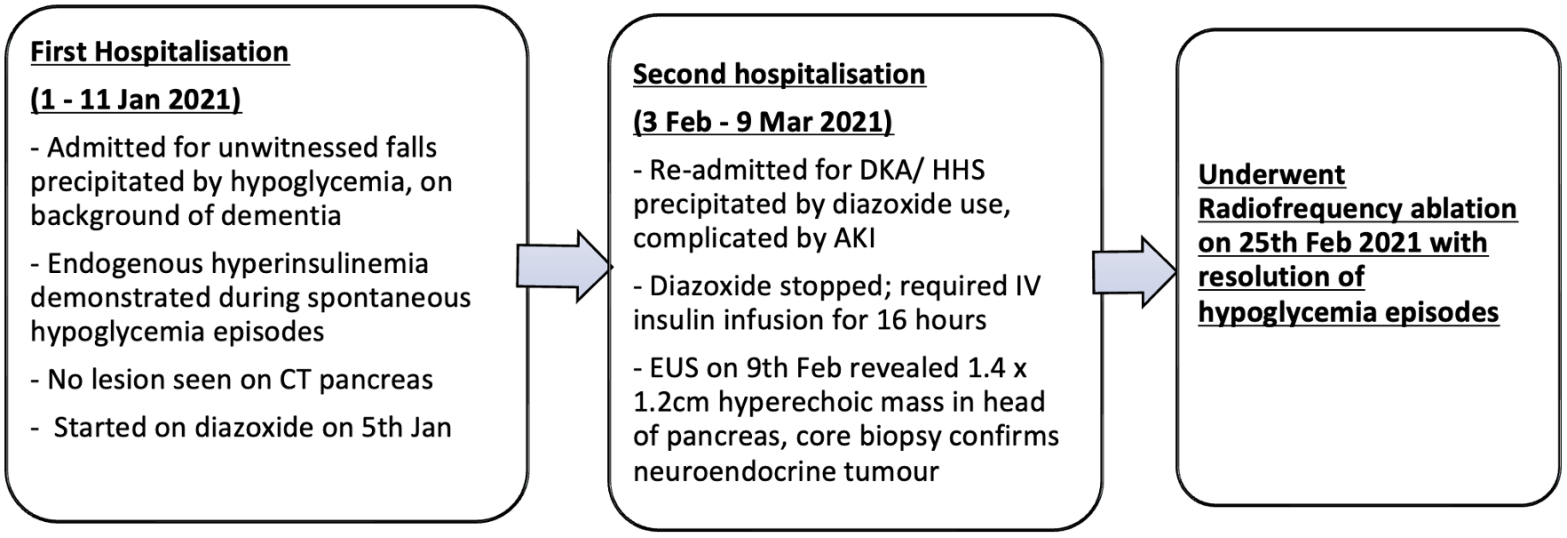
Timeline detailing patient's progress from diagnosis, followed by diazoxide-induced DKA/HHS, and eventual management with EUS-RFA.

She has had no clinically significant recurrences up until 4 years post-procedure (at the time of writing) (**Figure 3**).

## 4 Discussion

Surgical removal of the tumor is the treatment of choice for insulinoma to ablate hyperinsulinemic hypoglycemia. However, surgery is associated with a considerable risk of morbidity and mortality. Medical therapy to control symptomatic hypoglycemia should be considered in patients who are not candidates for or refuse surgery or who have unresectable metastatic disease. Other therapeutic strategies may include ablation in the form of radiofrequency ablation (4), high-intensity focused ultrasound ablation (5), and ultrasound assisted alcohol ablation (6). The patient, their family, and physicians should engage in shared medical decision-making with regard to treatment options, especially in geriatric patients who may not be able to make decisions on their own due to cognitive impairment and because they may be particularly vulnerable to post-surgical complications.

Dietary modification was initially advised, and the patient was encouraged to consume small frequent meals throughout the day to avoid symptomatic hypoglycemia, although this was challenging given her underlying cognitive impairment and poor appetite. In view of recurrent and persistent hypoglycemic episodes requiring a continuous intravenous dextrose infusion, she was started on diazoxide.

Diazoxide is a non-diuretic benzothiadiazine that inhibits insulin secretion by opening ATP-dependent potassium channels in pancreatic beta cells (8). The ability of diazoxide to increase hepatic glucose production and inhibit glucose uptake has also been reported (9). The therapeutic daily dosage in adults is 3-8 mg/kg divided into two or three equal doses. Typical treatment should be initiated with a daily dose of 150 to 200 mg given in two or three doses per day, with a clinically relevant therapeutic effect achieved in several days (10). Long-term treatment with diazoxide appears to be highly effective, with 59% of patients being symptom-free and only 38% with occasional symptoms. The common side effects of diazoxide may include fluid retention, hirsutism, weight gain, nausea, emesis, diarrhea, abdominal pain, headache, and rash.

The patient was unfortunately re-admitted with DKA/HHS precipitated by diazoxide 1 month after its initiation. There are only a few case reports (11–13) on hyperglycemic crises occurring following diazoxide therapy for treatment of endogenous hyperinsulinemia. These were elderly patients (age range 67 to 85 years old) who had either declined or were deemed unfit for surgical intervention. In these reports, the hyperglycemic crises had occurred in the setting of other contributing factors, including dehydration, infection, or new drugs (such as prednisolone), and after many years of being on diazoxide. The half-life of diazoxide varies between 24 to 36 hours, which may be further prolonged in the presence of renal impairment as it is renally excreted. Hence, there needs to be vigilance and close monitoring for side effects of diazoxide, especially in elderly patients with impaired renal function.

In the week leading up to her RFA procedure, the patient was also temporarily started on low-dose oral prednisolone in addition to intravenous dextrose infusion in view of the recurrent hypoglycemia episodes. Glucocorticoids work by increasing insulin resistance, stimulating gluconeogenesis, reducing glucose uptake, and inhibiting insulin synthesis. However, it has never attained a broader application in the treatment of hypoglycemia due to its wide range of adverse effects (9).

Other options that can be considered for refractory hypoglycemia include somatostatin analogs, such as octreotide (14) and lanreotide (15), although the efficacy is often less predictable, and they have also been reported to worsen hypoglycemia paradoxically by suppressing the release of glucagon and growth hormone.

Surgery is the treatment of choice for insulinomas with a high cure rate for localized disease, although it has a considerable risk of morbidity and mortality. However, in view of her advanced age and high operative risk, the patient in this case report was not deemed to be a suitable surgical candidate.

RFA is a treatment modality that has recently evolved but is not yet widely used. The first patient with insulinoma who was successfully treated with RFA was described in 2009 (16). RFA induces tumor mass thermal necrosis and hence can be potentially curative. It carries the risk of puncturing and thermal injury to the pancreas and adjacent critical structures. In a retrospective analysis (4) of 18 patients with pancreatic neuroendocrine tumors (including seven patients with insulinomas) who underwent ultrasound-guided RFA, a clinical response (defined by the resolution of hypoglycemia-related symptoms and normalization of glucose levels) was observed in all insulinoma cases within 24 hours of treatment. Overall, there were no major complications 48 hours post-procedure, and no clinically significant recurrences were observed during a mean follow-up of 8.7 ± 4.6 months (range 2 to 21 months). There is evolving evidence of RFA being a potential safe and feasible therapeutic modality for patients who refuse surgery (3) or who are at high surgical risk, or even as an alternative to surgery in selected patients with benign insulinoma. The limitations of RFA include an inability to evaluate tumor-free margins and the lack of additional prognostic markers of tumor aggressiveness from histological features (e.g., vascular/neural invasion) of the surgical tissue specimen.

This case highlighted the importance of a high index for suspicion for insulinomas, which is not only a rare condition but also may present atypically in elderly patients. Medical therapy for insulinoma with diazoxide, though effective, might be associated with side effects, especially in the elderly, and this requires close monitoring. Finally, the role of non-surgical options such as RFA in the treatment of insulinomas appears promising, although it needs to be further studied through larger multicenter studies with longer follow-ups.

## 5 Conclusion

This case describes a frail elderly lady with background dementia who was diagnosed with pancreatic head insulinoma. The initial choice of medical therapy with diazoxide resulted in a rare but previously reported complication of a hyperglycemic emergency (DKA/HHS). As she was a poor candidate for pancreatic surgery, she underwent successful EUS-guided RFA of the insulinoma with complete resolution of hypoglycemia and early normalization of glucose levels. EUS-RFA can be a successful definitive treatment option for insulinoma in suitable patients who have contraindications for pancreatic surgery, bearing in mind its limitations.

## Data Availability

The original contributions presented in the study are included in the article/supplementary material. Further inquiries can be directed to the corresponding author.
